# Synonymous Rare Arginine Codons and tRNA Abundance Affect Protein Production and Quality of TEV Protease Variant

**DOI:** 10.1371/journal.pone.0112254

**Published:** 2014-11-26

**Authors:** Jie Fang, Lingling Zou, Xuan Zhou, Beijiu Cheng, Jun Fan

**Affiliations:** Key Laboratory of Crop Biology of Anhui Province, School of Life Science, Anhui Agricultural University, Hefei, Anhui, PR China; University of Edinburgh, Western General Hospital, United Kingdom

## Abstract

It has been identified that a TEV protease (TEVp) variant, TEVp^5M^, displays improved solubility. Here, we constructed fifteen TEVp^5M^ variants with one or more of six rare arginine codons in the coding sequence replaced with abundant *E. coli* arginine codons. These codon variants expressed in either *E. coli* BL21 (DE3) or Rossetta (DE3) showed different solubility and activity. Supply of rare tRNAs increased the tendency of certain codon variants to form insoluble aggregates at early induction stage, as determined by the fused S-tag. About 32% increase in soluble protein production of M5 variant with four synonymously mutated arginine codons was identified in Rossetta (DE3) cells using GFP fusion reporter, comparable to that of TEVp^5M^. After purification, two other codon variants from both *E. coli* strains exhibited less activity than TEVp^5M^ on cleaving the native or modified recognition sequence incorporated between GST and *E. coli* diaminopropionate ammonialyase by enzyme-coupled assay, whereas purified M5 variant showed activity similar to the TEVp^5M^. Supply of rare tRNAs caused the decrease of activity of TEVp^5M^ and M5 by about 21%. Our results revealed that engineering of highly soluble TEVp variants can be achieved by the combined mutations of amino acid residues and optimization of specific rare codons, whereas simple augment of rare tRNAs abundance resulted in partial loss of activity.

## Introduction

As the host, *Escherichia coli* is fundamentally important in the manufacture of a wide range of biotechnological and biomedical products, owing to its easy culture, fast growth, simple fermentation and easy genetic manipulation [1]. High production of recombinant protein in *E. coli* is a combination of several factors such as efficiency of transcription, mRNA stability, mRNA folding, codon usage, protein solubility and folding [2]. So, the strategies for optimizing expression greatly vary from gene to gene and are often determined by trial and error.

In *E. coli*, tRNA levels for rare codons AGG, AGA, AUA, CCC, CUA and GGA restrain high-level production of the recombinant proteins [3]. Codon optimization could enhance soluble protein production [4],[5], but sometimes it also impaired protein folding and decreased activity [6],[7]. Similarly, supply of deficient tRNAs increased expression levels of recombinant proteins, but decreased soluble production of some recombinant proteins [8]. So far, obtaining well-folded recombinant proteins with high yield remains a major challenge, as yield, solubility and conformational quality of soluble proteins could not be simultaneously optimized in *E. coli* [9].

Fusion tags are frequently applied for improving protein solubility and facilitating rapid purification by affinity chromatography. To avoid potential interference with biological activity or crystallization, the fused tags need to be removed, which is often achieved by a specific protease that recognizes and cleaves the engineered sequence between the tag and target protein [10]. Tobacco etch virus protease (TEVp) has been identified as a tool, owing to its stringent recognition sequence. Production of wild type TEVp or the mutant TEVpS219V with inhibition of auto-cleavage in *E. coli* is limited due to its low solubility [10]. Previously, we developed a novel method for quantitatively measuring in vivo and in vitro activity of TEVp using a designed fusion protein as substrate [11], and engineered a variant of TEVp (TEVp^5M^) with five mutated amino acid residues that displayed improved protein solubility and thermo-stability [12].

There are six rare arginine codons (R49, R50, R80, R101, R105 and R159) in the TEVp coding sequence. With the assistance of solubility enhancer maltose-binding protein (MBP), a TEVp variant with the synonymous mutations of rare codons for R49 and R50 is found to display an enhanced soluble production in *E. coli* BL21(DE3) [13]. Here, we further demonstrated that synonymous mutations of rare arginine codons and increment of rare tRNAs affected protein production and quality of TEVp^5M^.

## Materials and Methods

### Bacterial strains, plasmids and reagents

*E. coli* strains DH5α, BL21(DE3), BL21(DE3)plysS and Rosetta(DE3), the plasmids pETDuet-1 and pET-22b, and the FRETWorks S-tag Assay Kit were purchased from Novagen (Madison, WI). Reagents used in plasmid construction, protein expression, and site-directed mutagenesis were bought from Takara (Dalian, China). The affinity matrix nickel-nitrilotriacetic acid (Ni–NTA) superflow is a product of Qiagen (Chatsworth, CA). The YM-10 membrane was supplied by Amicon (USA). Pyridoxal 5′-phosphate and DL-α,β-diaminopropionate (DL-DAP) were from Sigma (USA). DNA sequencing was performed by Invitrogen (Shanghai, China).

### Site-directed mutagenesis and plasmids construction

The plasmids such as pET28-TEVp^5M^, pET28-GFP and pGST-DAL were constructed previously [12]. Synonymous mutations of the six arginine codons in TEVp^5M^ coding sequence were conducted by PCR amplification, using pET28-TEVp^5M^ as the template. The primers were listed in supplementary Table S1. Each mutated gene was cut with *Nco* I and *Xho* I, and inserted into *Nco* I/*Sal* I sites of pET28-GFP plasmid. The mutated gene encoding M3 or M10 variant was also inserted into *Nco* I/*Xho* I sites of pETDuet-1 vector. The inserted gene in pGST-DAL plasmid encodes the fusion protein GST-tevS1-DAL containing His6-tagged GST, a natural TEVp cleavage site and *E. coli* diaminopropionate ammonia-lyase (DAL). Two amino acid residues glycine and serine in the TEVp recognition sequence ENLYFQ-GS were replaced by aspartic acid and glycine. The fusion protein containing the modified TEVp recognition sequence was named as GST-tevS2-DAL. The sequence ENLYFQ-DG is weakly recognized by TEVp [14]. All the mutations were identified by DNA sequencing.

### Detection of the recombinant proteins

Except where noted, production and extraction of recombinant proteins in this study were conducted as follows. *E. coli* BL21(DE3) cells expressing the TEVp^5M^ or codon variant were cultured overnight at 37°C in 5 ml of Luria–Bertani (LB) medium, diluted to 5-fold and grown at 37°C. When OD_600_ reached about 0.5, cells were induced by 0.5 mM of IPTG. After culturing at 28°C for 12 h, cells in 5 ml of LB medium were collected by centrifugation and disrupted in 0.8 ml of buffer A (20 mM Tris/HCl, pH 8.0, 100 mM NaCl). Pellets were washed three times with buffer A, solubilized by 0.2 ml of 8 M urea, and centrifuged to remove the precipitants. About 40 µg and 10 µg of proteins in supernatants and pellets respectively were analyzed by SDS-PAGE or Western blotting using mouse anti-His6 antibody. Protein concentration was determined by Coomassie brilliant blue G250, using bovine serum albumin as standard. The absorption of 8 M urea solution was applied as control and subtracted.

Recombinant codon variant or that fused with the bacterially codon-optimized emerald green fluorescent protein (EmGFP) were expressed in either *E. coli* BL21(DE3) or Rosseta (DE3) cells. Three colonies were selected randomly and the fluorescence of cells, soluble fractions and pellets were measured by an F-4500 fluorescence spectrometer (Hitachi, Japan). Excitation and emission was conducted at 488 nm and 515 nm. Cells harboring pET28-TEVp^5M^ were used as the control and cell fluorescence was subtracted.

The S-tagged TEVp^5M^ or selected codon variant were overexpressed in *E. coli* BL21(DE3) plysS cells and induced for 10, 20, 30, 40, and 50 min respectively at 37°C. Soluble protein production was determined by measuring the fused S-tag amount, according to Novagen's S•Tag System protocol. The reaction mixture contained 20 µl of FRET assay buffer, 2 µl of FRET ArUAA substrate, 5 µl of S-tag grade S-protein, and 153 µl of sterile deionized water. Reactions were initiated by adding 20 µl of soluble fraction, kept 5 min in the dark, and quenched immediately by adding 20 µl of stop solution. The solution was diluted to 2 ml and recorded fluorescence value at excitation and emission wavelengths of 495–530 nm with an F-4500 fluorescence spectrometer [12]. Three colonies were cultured and protein amounts were analyzed. Cells harboring the plasmid pET-22b were used as the control and fluorescence signal was subtracted.

### Purification of recombinant proteins

*E. coli* cells overexpressing the TEVp variant, or fusion protein as TEVp substrate were collected, re-suspended in buffer B (50 mM sodium phosphate, 300 mM NaCl and 10 mM imidazole, pH 8.0), sonicated and centrifuged. Recombinant proteins in supernatants were purified by Ni-NTA resin according to QIAexpressionist protocol. After cleavage of purified two fusion proteins by TEVp^5M^, the DAL was released from Ni-NTA using buffer B. Purified proteins were concentrated and exchanged with buffer A.

### Coupled assay of TEVp activity

Activity of purified DAL as the fusion partner or without the fusion tag was measured using DL-α,-β-diaminopropionate as a substrate to react for 5 min at 37°C. Amount of pyruvate was measured with 2,4-dinitrophenylhydrazine. Absorbance at 520 nm was recorded in a U-2001 spectrometer (Hitachi, Japan) [11]. The purified GST-tevS1-DAL and extracted (or purified) codon variant with the mass ratio of 5∶1 was reacted at 30°C for 1 h. After reaction, activity of released DAL was analyzed. Activity of purified TEVp^5M^ and the three codon variants for cleaving modified sequence was analyzed after reacting at 25°C for 6 h. Purified GST-tevS2-DAL and TEVp construct were mixed with the mass ratio of 10∶1 [15]. The DAL activity for the protein substrate mixed with heat-inactivated TEVp^5M^ in soluble fraction or purified form at 100°C for 5 min was applied as the control and subtracted.

## Results

### Construction of the synonymously mutated TEVp^5M^

Since TEVp^5M^ is more soluble than the TEVpS219V variant [11], we introduced a series of synonymous mutations into six rare arginine codons in TEVp^5M^ coding sequence converting them to abundant arginine codons found in *E. coli*. The codon variants constructed include M1–M4 with one or two rare arginine codons being replaced with the optimized arginine codons, and M5–M15, which combined to the mutations in M1–M4 with additional codons (Table 1). The constructed plasmids for expressing the codon variants contain double His6-tag at both ends, or the N-terminal His6-tag and C-terminal GFP harbored the T7 promoter, a ColE1 origin of replication, and a kanamycin resistance marker. The other constructed vectors for expressing the variants with the N-terminal His6-tag and C-terminal S-tag contain the T7 promoter, a p15A origin of replication, and a chloramphenicol acetyltransferase gene.

**Table 1:** The synonymous codon substitution in the TEVp^5M^ coding sequence.

Variant	Sequence					
	R49	R50	R80	R101	R105	R159
TEVp^5M^	AGA	AGA	AGG	AGA	AGG	AGA
M 1	CGT	CGC	AGG	AGA	AGG	AGA
M 2	AGA	AGA	AGG	CGT	CGC	AGA
M 3	AGA	AGA	CGT	AGA	AGG	AGA
M 4	AGA	AGA	AGG	AGA	AGG	CGT
M 5	CGT	CGC	AGG	CGT	CGC	AGA
M 6	CGT	CGC	CGT	AGA	AGG	AGA
M 7	CGT	CGC	AGG	AGA	AGG	CGT
M 8	AGA	AGA	CGT	CGT	CGC	AGA
M 9	AGA	AGA	AGG	CGT	CGC	CGT
M 10	AGA	AGA	CGT	AGA	AGG	CGT
M 11	CGT	CGC	CGT	CGT	CGC	AGA
M 12	CGT	CGC	CGT	AGA	AGG	CGT
M 13	CGT	CGC	AGG	CGT	CGC	CGT
M 14	AGA	AGA	CGT	CGT	CGC	CGT
M 15	CGT	CGC	CGT	CGT	CGC	CGT

### Qualitative analysis of the expressed codon variants

**Figure 1:**
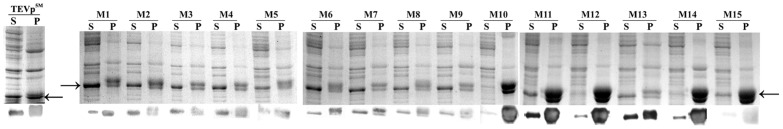
Expression of TEVp^5M^ and codon variants in *E. coli* BL21(DE3). About 40 µg of total proteins in soluble fractions, and 10 µg of proteins in pellets solubilized by 8 M urea were separated by SDS-PAGE. Variant names are indicated above each gel lane. Arrows indicate positions of TEVp^5M^ or the codon variants. Specific bands detected by Western blotting are displayed at bottom.

SDS-PAGE and Western blot analyses showed that the expression levels of fifteen codon variants in soluble and insoluble fractions varied. Five variants including M10, M11, M12, M14 and M15 were expressed mainly as inclusion bodies, and other variants were expressed in soluble fractions and pellets (Fig. 1). The results suggested that change of certain arginine codons with abundant ones affected protein production and folding of TEVp^5M^ in *E. coli*.

### Quantitative analysis of the expressed codon variants

**Figure 2:**
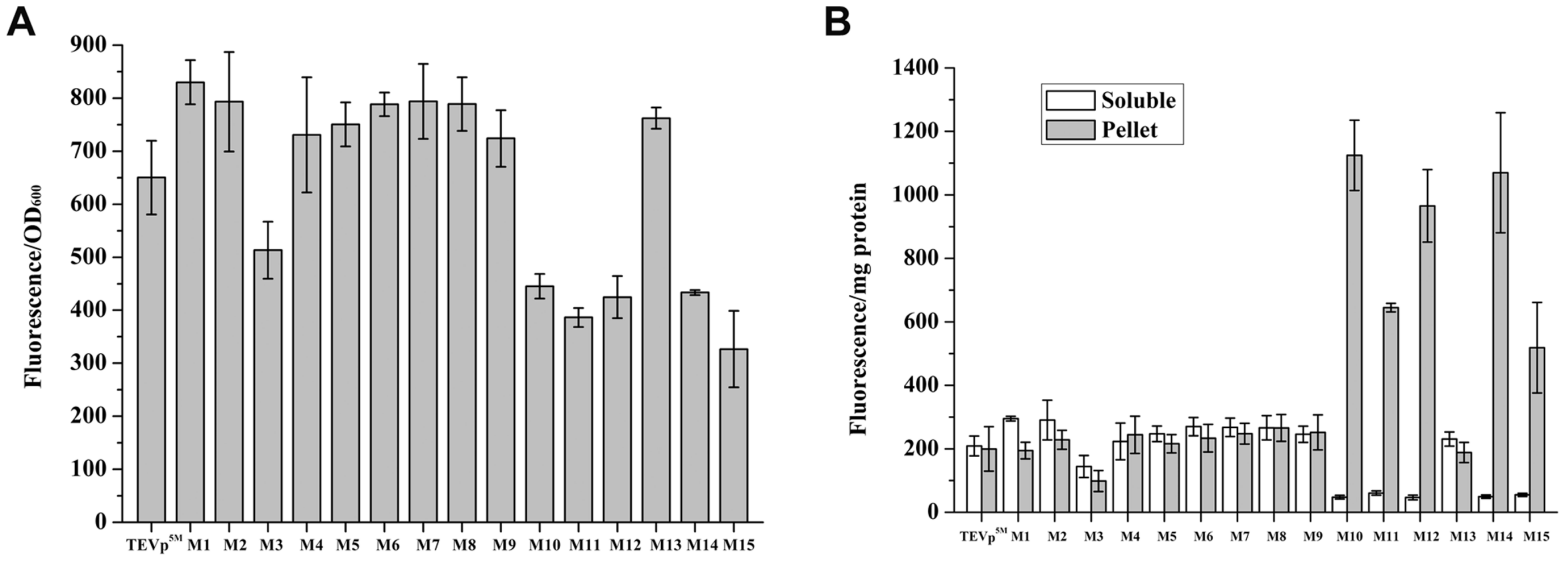
Relative GFP fluorescence assay for GFP fused TEVp^5M^ and fifteen codon variants expressed in *E. coli* BL21(DE3). (A) Relative fluorescence intensities of cells expressing the codon variant fused with GFP. (B) Relative fluorescence intensities of soluble fractions and pellets.

When expressed in the fusion form with the GFP reporter, nine codon-optimized variants emitted slightly higher cell fluorescence than TEVp^5M^, whereas six other variants displayed less fluorescence (Fig. 2A). The M1 mutant with synonymous mutations of AGA-AGA clusters of R49 and R50 showed the highest value of relative fluorescence intensity, corresponded with the other report [13]. Six out of the fifteen variants showed different response between their cell-based fluorescence with the fluorescence from soluble fractions, with extra fluorescence trapped in insoluble pellets (Fig. 2A and 2B). Because this method allows the quantification of cell-based fluorescence, which is contributed by protein-emitted fluorescence from insoluble aggregates and soluble form, the solubility of engineered TEVp can thus be evaluated.

### Effects of increasing rare tRNAs on production of the selected codon variants

**Figure 3:**
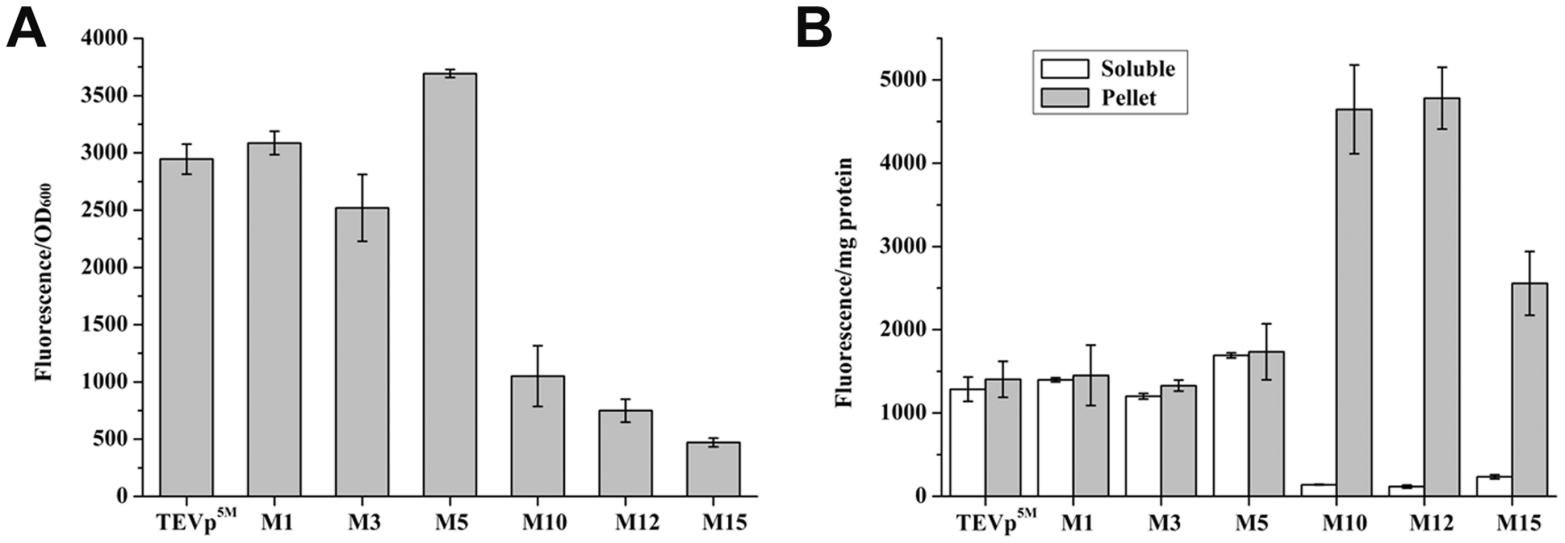
GFP fluorescence assay for GFP fused TEVp^5M^ and the selected codon variants expressed in the Rossetta (DE3) strain. (A) Relative cell fluorescence per OD_600_. (B) Relative fluorescence intensities of soluble fractions and pellets.

In addition to rare AGA and AGG codons for arginine, there are other four rare codons in *E. coli*. We noted these rare codons, including four AUA (isoleucine), one CUA (leucine), one CCC and three GGA (glycine), present in the TEVp coding sequence (https://www.addgene.org/8827/sequences/). *E. coli* strain Rosetta (DE3) harbors the plasmid pRARE that supplies tRNAs for all of the six rare codons. To detect effect of rare tRNAs on enhancing protein expression level, we analyzed protein solubility of six codon variants in Rosetta (DE3). These variants displayed different soluble expression levels in BL21(DE3). As a result, soluble expression levels of all six variants tested were higher in Rosetta (DE3) than in BL21(DE3) (Fig. 2A and 2B, Fig. 3A and 3B). Comparing with TEVp^5M^, M1 and M5 were more soluble, while other four variants including M3, M10, M12 and M15 were less soluble. In addition, M1 emitted highest fluorescence in BL21(DE3), it became less fluorescent than M5 in Rosseta (DE3), indicating the rare tRNAs may preferably promote M5 experssion. The variant M5 contains four synonymously mutated codons at R49, R50, R101 and R105. About 32% increase in soluble yield of M5 variant was determined in Rossetta (DE3), compared to 15% in the BL21(DE3). Similarly, several other codon variants expressed as insoluble aggregates also emitted stronger fluorescence by co-expressing rare tRNAs.

### Soluble expression level of the codon variants induced at initial induction

**Figure 4:**
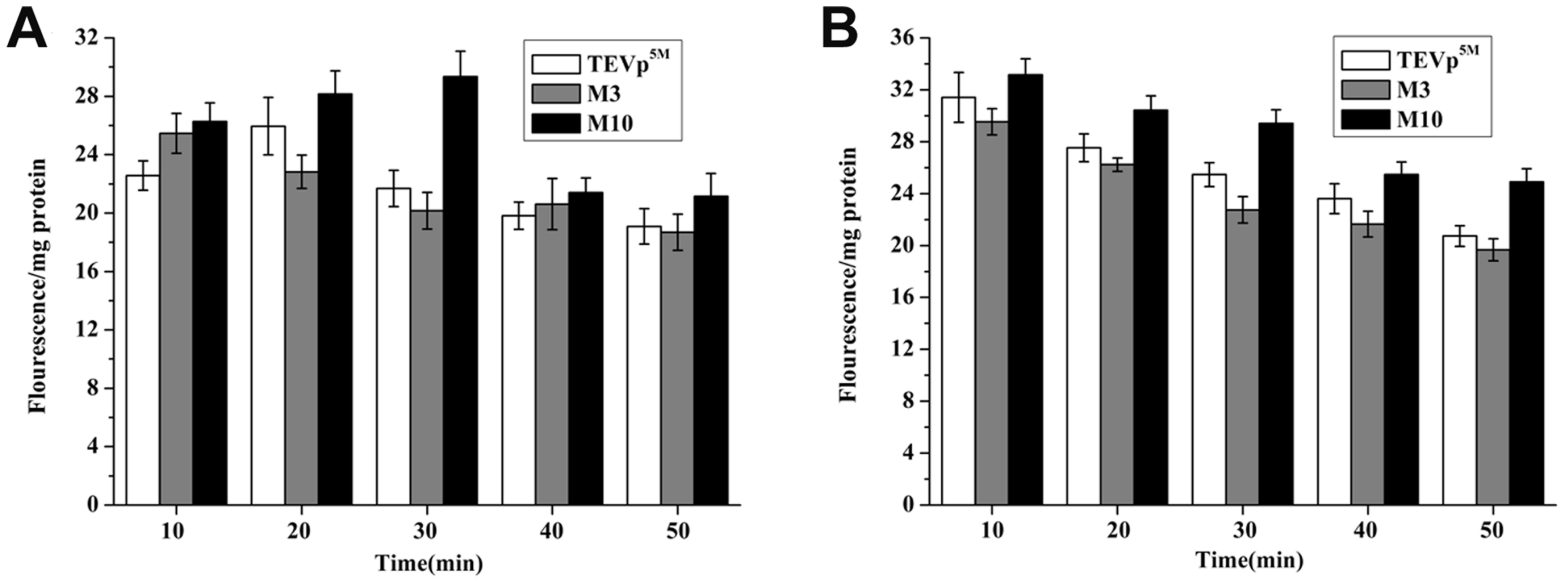
Soluble expression levels of S-tagged TEVp^5M^ and some codon variants at early induction stage. (A) Relative fluorescence of proteins expressed in *E. coli* BL21(DE3). (B) Relative fluorescence of proteins expressed in Rosseta (DE3).

By detecting the fused S-tag amount, soluble protein production of TEVp^5M^, M3, and M10 were analyzed following initial induction. One arginine codon at R80 was synonymously mutated in M3, and another mutated arginine codon at R159 was combined in M10. The soluble expression levels of the two codon variants were affected by simple augmentation of rare tRNAs, based on cell fluorescence using GFP reporter (Fig. 2A and 3A). The S-tagged variants were expressed in a different *E. coli* strain BL21(DE3)plysS to inhibit background expression. The maximum expression levels of soluble TEVp^5M^, M3 and M10 were detected after induction at 37°C at 20 min, 10 min, and 30 min respectively. With induction time prolonged more than 30 min, soluble amounts of all three TEVp constructs were decreased (Fig. 4A). When the three proteins were expressed in *E. coli* Rosetta (DE3), their soluble expression levels were all decreased after 10 min induction (Fig. 4B), suggesting that the supplied rare tRNAs increased the tendency of certain codon variants to form insoluble aggregates at 37°C. The reason for the difference among codon variants were possibly related to the different position of rare codons in the coding sequence that altered folding process during translation thus the protein solubility, as well as the aggregation with the existing TEVp proteins.

### Activity of TEVp^5M^ and fifteen variants in the crude extracts

**Figure 5:**
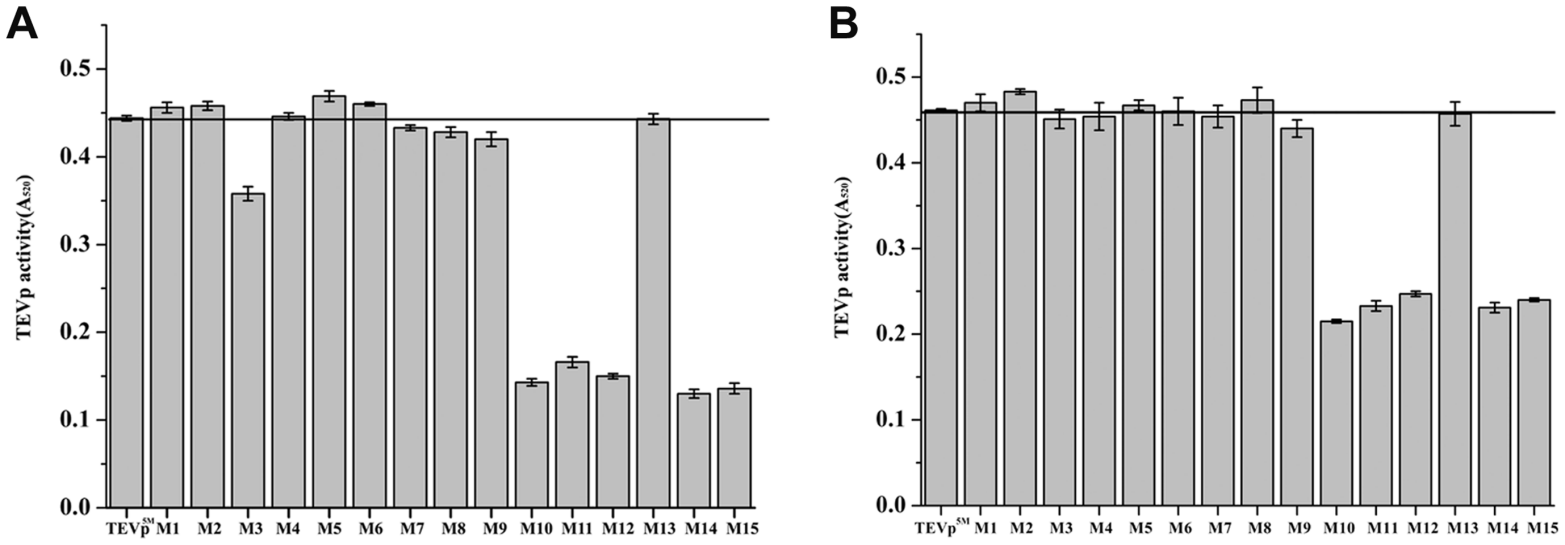
Activity of soluble TEVp^5M^ and fifteen codon variants. Cleavage activity of the constructed TEVp^5M^ and variants expressed in *E. coli* strains BL21(DE3) (A) and Rosseta (DE3) (B) respectively. The fusion protein GST-tevS1-DAL was used as the substrate. After cleavage, the released DAL activity was measured, as described in Materials and Methods.

To test sensitivity of the DAL reporter system, we analyzed the activity of DAL. The specific activities of DAL from purified GST-tevS1-DAL and GST-tevS2-DAL were about 2.1 and 2.2 U/mg protein respectively, whereas those of purified DAL by cleaving the fusion protein and removing the fusion tag were approximately 68 and 73 U/mg protein. This character is sufficient for sensitively analyzing TEVp activity by the enzyme-coupled assay. The four variants M1, M2, M5 and M6 expressed in BL21(DE3) showed slightly higher activity than TEVp^5M^ for cleaving protein substrate GST-tevS1-DAL, two variants M4 and M13 exhibited similar cleavage efficiency, and other variants were less active than TEVp^5M^ (Fig. 5A). Co-expression of rare tRNAs increased activity of certain codon variants (M10, M11, M12, M14 and M15) that were expressed mainly as inclusion bodies, but did not increase activity of other variants significantly (Fig. 5B). The different cleavage activity of the soluble codon variants expressed in two *E. coli* strains was detected respectively by SDS-PAGE (Fig. S1). Among the constructs, M5 expressed in BL21(DE3) and M2 expressed in Rosetta(DE3) showed the highest activity, even soluble production of M5 was less than M1 in BL21(DE3). M2 contains two synonymously mutated codons at R101 and R105. These astonishing results suggested that, even with identical amino acid sequences, protein quality of certain codon variants was decreased upon enhancing protein production by the increased levels of rare tRNAs.

### Activity of purified TEVp^5M^ and the selected variants

**Figure 6:**
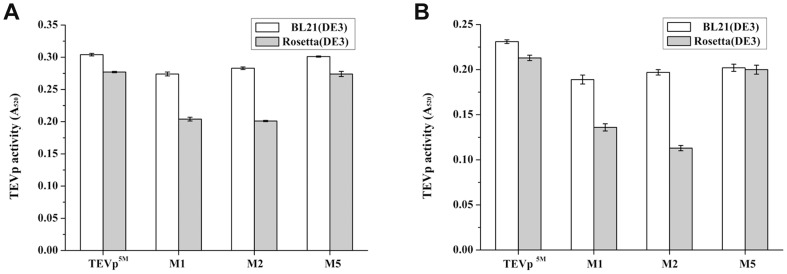
Substrate specificity of the purified TEVp^5M^ and selected codon variants. Activity of the TEVp^5M^ and selected three variants expressed in *E. coli* BL21(DE3) or Rosseta (DE3) using protein substrates GST-tevS1-DAL (A) and GST-tevS2-DAL (B).

The three variants M1, M2 and M5 that were more soluble and active than TEVp^5M^ were selected for further purification. The GST-tevS1-DAL coupled assay showed that activity of purified variants M1 and M2 from *E. coli* BL21(DE3) was slightly less than that of the TEVp^5M^, while M5 displayed similar activity as TEVp^5M^ (Fig. 6A). Supply of rare tRNAs decreased specific activities of all four TEVp constructs, especially for M2 (Fig. 6B). The activities of TEVp^5M^ and M5 were also decreased by about 21%. The different cleavage efficiency of purified variants from two *E. coli* strains was further confirmed by SDS-PAGE (Fig. S2). The results suggested that the yield and quality of soluble TEVp^5M^ variants are somewhat contradictory, and optimization of both parameters can be challenging for proteins recombinantly expressed in *E. coli*.

## Discussion

Previously, we have overexpressed three TEVp variants including the TEVpS219V in *E. coli* Rossetta (DE3) and obtained about 80 mg pure protein from 1L LB culture [11]. As a comparison, only about 3.2 mg of bacterially codon-optimized TEVpS219V was purified from 100 ml LB medium with the assistance of MBP and in vivo self-cleavage [16]. The exact mechanism for the dramatic improvement of yield was unclear, and high-level production of soluble protein in *E. coli* is affected by several factors including rare codons, transcriptional and translational rates, mRNA structure and stability. In this study, we investigated the possible effect of codon frequency to the production of active TEVp^5M^, using a combination of six rare arginine codons to generate a collection of fifteen variants with identical protein sequences. When all six rare arginine codons were substituted with abundant codons, the variant M15 was expressed mainly as inclusion bodies, as detected by SDS-PAGE and Western blotting. This partly explained why the codon-optimized TEVpS219V was not produced at high yield in *E. coli*.

To detect protein folding in vivo, we applied EmGFP reporter since it emits stronger fluorescence than other variants [17], and cell fluorescence is correlated with soluble protein amounts using either spectrofluorimetry or flow cytometry [18],[18,19]. We found that GFP in pellets also contributed to cell fluorescence. GFP is active even within protein aggregation [20], but the aggregated GFP gives rise to partial loss of fluorescence [21]. Synonymous mutated rare-to-abundant codons resulted in increase of protein synthesis but in turn affected GFP folding [6], thus, it cannot be assumed that GFP fluorescence in pellet is maintained. Nonetheless, the cell fluorescence combined the signal from both soluble and insoluble form of TEVp-EmGFP thus provided rapid evaluation of overall performance of the designed TEVp codon variants in *E. coli*. However, quantitative protein amount in pellets could not be assessed reliably by this method, owing to the effect of target protein on GFP folding. A more sensitive approach such as using split GFP reporter [22], should be exploited.

Because GFP requires a long lag phase (95 min) to form the chromophore [23], we used S-tag as the fusion reporter to detect soluble expression level of the TEVp variants at early time points upon induction. As a 15-amino-acid peptide, S-tag can be used to quantitatively detect protein amount. Moreover, S-tag has less effect on protein folding than GFP, though it is not suitable for analyzing protein amount in pellets because the denaturants such as urea inhibit the S-tag complementing S-protein to reconstitute RNase S [24].

Even though expression levels of certain codon variants were elevated in *E. coli*, improvement of enzymatic activity is more desirable. The current study demonstrated that the co-expressed rare tRNAs can enhance soluble protein production, but not efficiently improve protein quality. Recently, it was proposed that screening codon variants is an effective approach to augmenting soluble expression level [7]. However, we discovered that M2 expressed in both *E. coli* strains displayed higher activity in the crude extracts but less activity in the purified form than TEVp^5M^. In contrast, M5 showed similar activity to TEVp^5M^ in the purified form, but higher activity in the crude extract. Therefore, it is essential to evaluate protein production and quality of the codon variants by analyzing the activity in supernatants and purified form.

In conclusion, we confirmed that the synonymous rare-to-abundant arginine codon substitutions and tRNA abundance affected the active production and folding of TEVp^5M^. Supply of rare tRNAs increased soluble production, but concomitantly caused partial activity loss for certain codon variants. The correlation between in vivo protein expression, solubility and folding, and cleavage efficiency provided a consistent view of the effect of synonymously mutations on the soluble production and function of the sixteen TEVp constructs.

## Supporting Information

Figure S1 **SDS-PAGE analysis of the fusion protein GST-tevS1-DAL cleaved by soluble TEVp^5M^ and fifteen codon variants.** Proteins were overexpressed in either BL21(DE3) (A) or Rosseta (DE3) (B). His6-tagged GST-tevS1-DAL and cleaved products were indicated by arrows. Released DAL with glycine as the first amino acid residue was labeled as G-DAL. The His6-tagged GST with partial TEVp recognition sequence was labeled as GST.
(TIF)

Figure S2 **Cleavage of two protein substrates by purified TEVp^5M^ and three codon variants detected by SDS-PAGE.** The protease mutants were purified from *E. coli* BL21(DE3) for cleaving GST-tevS1-DAL (A) or GST-tevS2-DAL (B). The cleavage of two proteins substrates by purified variants from Rosseta (DE3) were also displayed (C and D). Protein substrate, cleaved products and TEVp constructs were indicated by arrows. After cleavage of GST-tevS2-DAL, released DAL with aspartic acid as the first amino acid residue was labeled as D-DAL.
(TIF)

Table S1 **Primers used in this study.**
(DOC)
