# Supplementary figures and images for: Synonymous Rare Arginine Codons and tRNA Abundance Affect Protein Production and Quality of TEV Protease Variant
Source: PLoS One. 2014 Nov 26;9(11):e112254. doi: 10.1371/journal.pone.0112254 (PMC4245098; doi:10.1371/journal.pone.0112254)

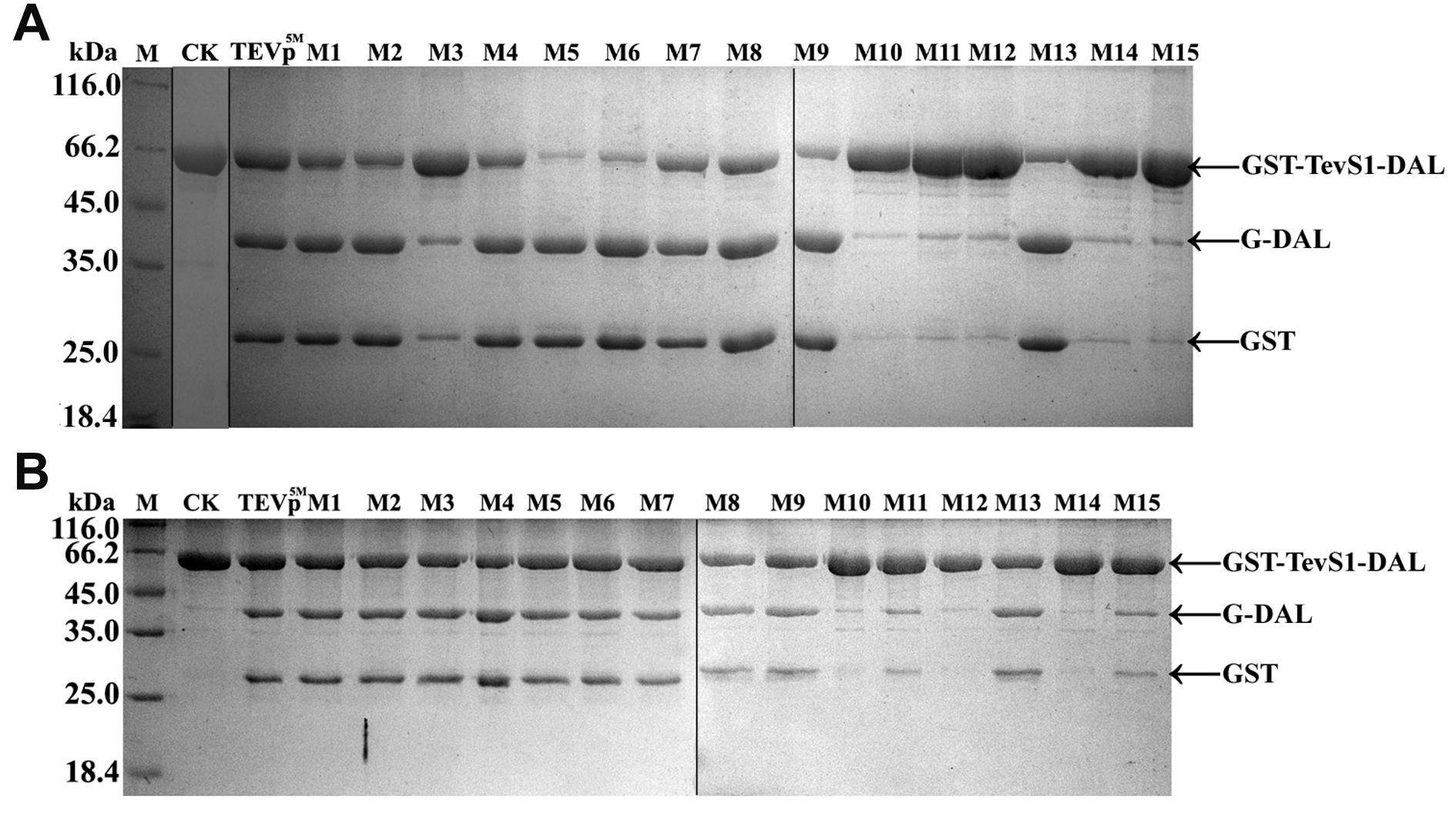

Supplement: Figure S1 — SDS-PAGE analysis of the fusion protein GST-tevS1-DAL cleaved by soluble TEVp5M and fifteen codon variants. Proteins were overexpressed in either BL21(DE3) (A) or Rosseta (DE3) (B). His6-tagged GST-tevS1-DAL and cleaved products were indicated by arrows. Released DAL with glycine as the first amino acid residue was labeled as G-DAL. The His6-tagged GST with partial TEVp recognition sequence was labeled as GST. (TIF) [file pone.0112254.s001.tif]

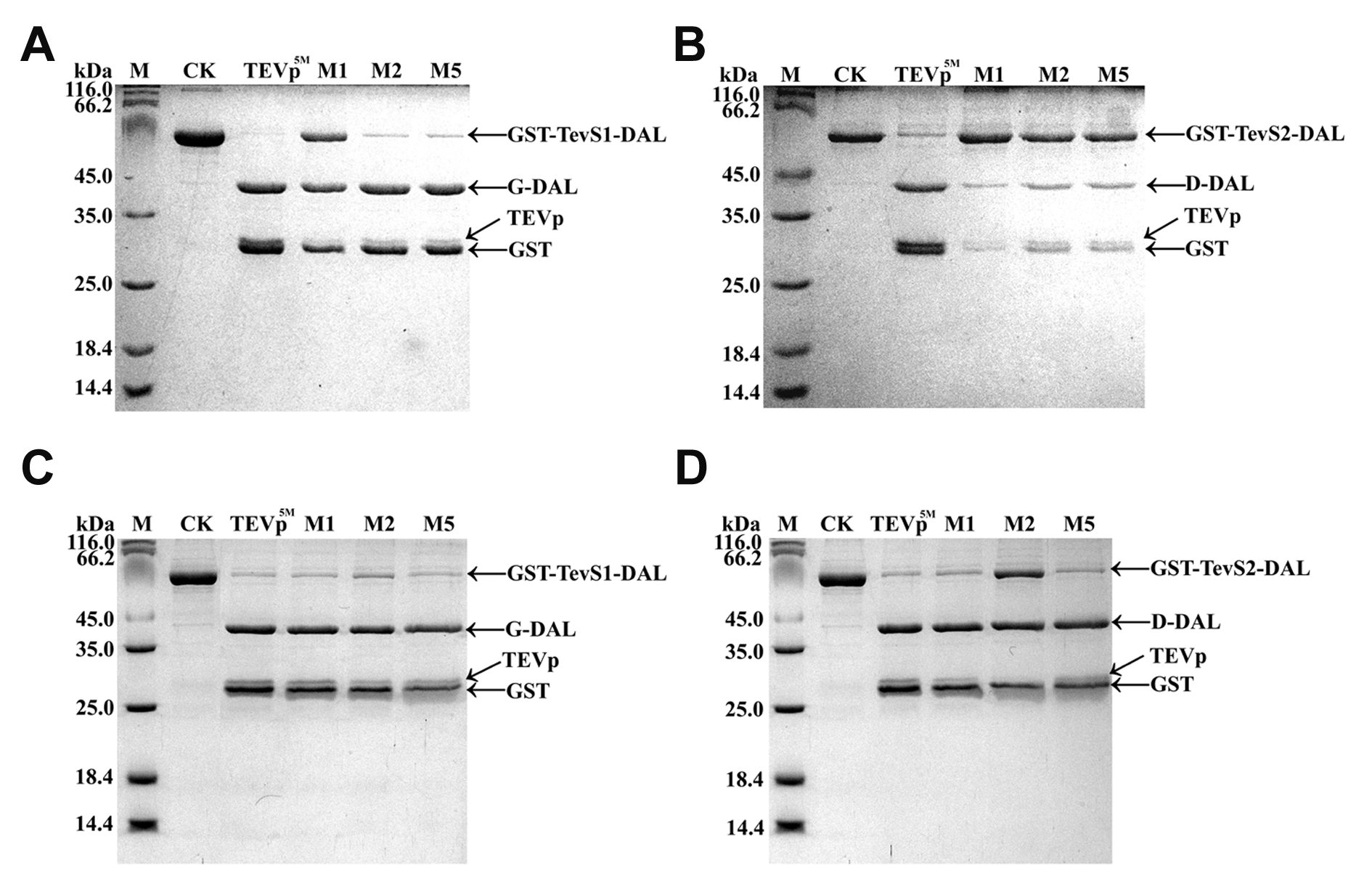

Supplement: Figure S2 — Cleavage of two protein substrates by purified TEVp5M and three codon variants detected by SDS-PAGE. The protease mutants were purified from E. coli BL21(DE3) for cleaving GST-tevS1-DAL (A) or GST-tevS2-DAL (B). The cleavage of two proteins substrates by purified variants from Rosseta (DE3) were also displayed (C and D). Protein substrate, cleaved products and TEVp constructs were indicated by arrows. After cleavage of GST-tevS2-DAL, released DAL with aspartic acid as the first amino acid residue was labeled as D-DAL. (TIF) [file pone.0112254.s002.tif]
