# Supplementary material for: Synonymous Rare Arginine Codons and tRNA Abundance Affect Protein Production and Quality of TEV Protease Variant
Source: PLoS One. 2014 Nov 26;9(11):e112254. doi: 10.1371/journal.pone.0112254 (PMC4245098; doi:10.1371/journal.pone.0112254)
Supplement: Table S1 — Primers used in this study. (DOC) [file pone.0112254.s003.doc]

Table S1

| **Oligonucleotide** | **Length** | **Sequence(5′→3′)** |
| --- | --- | --- |
| tvm1 | 38 | CAAACAAGCACTTGTTTCGTCGCAATAATGGAACACTG |
| tvm2 | 38 | CAGTGTTCCATTATTGCGACGAAACAAGTGCTTGTTTG |
| tvm3 | 39 | CAACGTGAAGAGCGCATTTGTCTTGTGACAACCAACTTC |
| tvm4 | 39 | GCGCTCTTCACGTTGTGGCTCGCGAAATTTCAGCTTTTG |
| tvm5 | 31 | CTCGTTGATGGGCGTGACATGATAATTATTC |
| tvm6 | 31 | CATGTCACGCCCATCAACGAGGTGTTGTTGC |
| tvm7 | 30 | CAACTCGTGATGGGTTCATTGTTGGTATAC |
| tvm8 | 31 | GAACCCATCACGAGTTGATACTAATGGACTG |
| tevS2-1 | 25 | CTGAAAATACAGGTTTTCAGATCTC |
| tevS2-2 | 25 | GATGGTGTTTTCTCATTGAAGATTG |

The primes used in this study
